# Analysis of pharmacovigilance databases for spontaneous reports of adverse drug reactions related to substandard and falsified medical products: A descriptive study

**DOI:** 10.3389/fphar.2022.964399

**Published:** 2022-09-06

**Authors:** Kevin Pozsgai, Gergő Szűcs, Anikó Kőnig-Péter, Orsolya Balázs, Péter Vajda, Lajos Botz, Róbert György Vida

**Affiliations:** ^1^ Department of Pharmaceutics and Central Clinical Pharmacy, Faculty of Pharmacy, University of Pécs, Pécs, Hungary; ^2^ Institute of Bioanalysis, Medical School, University of Pécs, Pécs, Hungary

**Keywords:** adverse drug reactions, falsified and substandard medicine, counterfeit medicine, health consequences, pharmacovigilance

## Abstract

**Introduction:** The public health threat of substandard and falsified medicines has been well known in the last two decades, and several studies focusing on the identification of products affected and preventing consumption have been published. However, the number of these products reaching patients and causing health consequences and adverse drug reactions is not a well-researched area.

**Objectives:** Our aim was to identify and describe the characteristics of cases that are related to adverse drug reactions potentially originating from counterfeit medication using publicly available pharmacovigilance data.

**Methods:** A descriptive study was performed based on pharmacovigilance data retrieved from Individual Case Safety Reports (ICSRs) identified in the European Medicines Agency’s EudraVigilance and FDA Adverse Event Reporting System (FAERS) databases in April 2022 using selected MedDRA preferred terms: counterfeit product administered, product counterfeit, product label counterfeit, product packaging counterfeit, suspected counterfeit product, adulterated product, product tampering, and suspected product tampering. ICSRs were analyzed by age and gender, by year of reporting, region of origin, reporter’s profession, and severity of the outcome. The disproportionality method was used to calculate pharmacovigilance signal measures.

**Results:** A total of 5,253 cases in the FAERS and 1,049 cases in the EudraVigilance database were identified, generally affecting middle-aged men with a mean age of 51.055 (±19.62) in the FAERS and 64.18% of the cases between 18 and 65 years, while the male to female ratios were 1.18 and 1.5. In the FAERS database, we identified 138 signals with 95% confidence interval including sildenafil (*n* = 314; PRR, 12.99; ROR, 13.04; RRR, 11.97), tadalafil (*n* = 200; PRR, 11.51; ROR, 11.55; RRR, 10.94), and oxycodone (*n* = 190; PRR, 2.47; ROR, 2.14; RRR, 2.47). While in the EV data 31, led by vardenafil (*n* = 16, PRR = 167.19; 101.71–274.84; 95% CI, RRR = 164.66; 100.17–270.66; 95% CI, ROR = 169.47; 103.09–278.60; 95% CI, *p* < 0.001), entecavir (*n* = 46, PRR = 161.26, RRR = 154.24, ROR = 163.32, *p* < 0.001), and tenofovir (*n* = 20, PRR = 142.10, RRR = 139.42, ROR = 143.74, *p* < 0.001).

**Conclusion:** The application of pharmacovigilance datasets to identify potential counterfeit medicine ADRs can be a valuable tool in recognition of potential risk groups of consumers and the affected active pharmaceutical ingredients and products. However, the further development and standardization of ADR reporting, pharmacovigilance database analysis, and prospective and real-time collection of potential patients with health consequences are warranted in the future.

## Introduction

Substandard and falsified medicines are a global public health problem in every country, however, on a different scale. According to the World Health Organization (WHO), 1 in 10 medical products in developing countries is falsified (World Health Organization, 2017a; World Health Organization, 2017b). Other estimates related to the closed drug supply chain and in the developed countries say that it is less than 0.005% (EU legal supply chain) (EC impact assessment accompanying the proposal for the Falsified Medicines Directive, 2015). It can be noted that with potential economic and health consequences, the situation during the COVID-19 pandemic has worsened in most countries (Gnegel et al., 2020; Shiferie and Kassa, 2020; Tchounga et al., 2020; Farmer, 2021; Fittler et al., 2021). As there are limited and controversial data regarding the prevalence of substandard and falsified medicinal products and usually these data refer to the number of boxes or ratio of sales affected by these illegitimate maneuvers, the number of products actually reaching the consumers and patients is lacking (Ozawa et al., 2018; McManus and Naughton, 2020; Pisani et al., 2021). Generally, it can be seen in the literature that case studies have been published in the last 20 years in this area. Rahman et al. (2018), in 2018, reviewed the available literature and found 48 reported incidents, 56.3% of which occurred in developing countries and 43.7% in developed countries, affecting approximately 7,200 consumers and resulting in 3,604 deaths, published as 45 incidents from 1969 to 2016 period. In their work, they highlight that the quality of these reports only provided inadequate or conflicting data. Although Anđelković et al. (2017) published their prospective data collection tool to identify patients harmed by substandard and falsified medicines, their method has not been widely used yet. In many countries, the complete investigation of these cases is rare and related to forensic departments and laboratories. Clinical investigation and publication of affected products and health care damages can be impactful public health actions, as in a case series in Columbia, to protect consumers and raise awareness (Peña-Acevedo et al., 2020). The new pharmacovigilance legislation implemented in 2012 broadened the definition of adverse drug reactions and included the unlicensed use, abuse, and falsified medicines (“...adverse reactions in human beings arising from the use of the medicinal product from use outside the terms of the marketing authorization”). With the development and evolution of the pharmacovigilance databases and data transparency guidelines, these data have become available to the public and researchers as well (Juhlin et al., 2015; European Medicines Agency, 2017).

Therefore, our aim was to find out whether these databases contain cases related to substandard and falsified medicinal products and to describe the characteristics of cases reported as adverse drug reactions related to substandard and falsified medical products and submitted to the European and United States public pharmacovigilance databases.

## Materials and methods

A descriptive case series study was performed based on pharmacovigilance data. If multiple versions or dates were present for a case, the latest version was used for further analysis. Between January and April 2022, two major pharmacovigilance databases were reviewed: 1) European Medicines Agency (EMA EudraVigilance (European Union)) and 2) FDA Adverse Event Reporting System (FAERS) Public Dashboard (United States). Individual Case Safety Reports (ICSRs) were extracted, and adverse drug reactions potentially related to falsified and substandard medicines were summarized. Report was included if any of the search terms were present in it. ICSRs were analyzed by age and gender, by year of reporting, region of origin, reporter’s profession, and severity of the outcome.

### EudraVigilance

EudraVigilance (EV) is a web-based, publicly available system launched by the European Medicines Agency in 2012 designed to collect suspected adverse drug reactions for medicines authorized in the European Economic Area (EEA). Although the database has been in use since December 2001, we searched for ICSRs and line listings of ADRs from 2002 to 2022. The data are submitted by the national medicine regulatory authorities (which contain the reports from the public and healthcare professionals) and by pharmaceutical companies with marketing authorization (Dubrall et al., 2021; European Medicines Agency, 2022a).

### FAERS public database

The FAERS Public Dashboard is a publicly available web-based tool containing mandatory data reports from drug manufacturers and voluntary ADR reports from consumers and healthcare professionals (MedWatch, ADR reporting programs) mainly from the United States. Although public access is available since September 2017, the queries can be viewed from 1968 (US Food and Drug Administration, 2022).

### Search strategy

ADRs are coded using MedDRA, endorsed by the International Conference on Harmonization of Technical Requirements for Registration of Pharmaceuticals for Human Use (ICH), which is a clinically validated international medical terminology dictionary used by regulatory authorities in the pharmaceutical industry during the regulatory process, from pre-marketing to post-marketing activities, and for data entry, retrieval, evaluation, and presentation. MedDRA dictionary is organized by System Organ Class (SOC), divided into high-level group terms (HLGTs), high-level terms (HLTs), preferred terms (PTs), and finally into lowest level terms (LLTs). We performed our analysis at the PT level, generally used in real-world pharmacovigilance research (Brown et al., 1999; Geneva: Council for International Organizations of Medical Sciences (CIOMS), 2016).

According to the MedDRA classification system, we can differentiate potential cases related to substandard and falsified medical products with the non-specified and specified system organ class (SOC), like injury, poisoning and procedural complications, and product issues. The authors searched for potential predetermined Standardized MedDRA Queries (SMQs and sub-SMQs) but did not find any. Two publications were identified with preferred terms linked to SF medicines. One was the monitoring medicines project with twenty-four preferred terms that may be in connection with SF medicines; however, no published version of the PT was available (Pal et al., 2015). The other publication from Sardella et al. (2021) categorized the PTs related to product issues (SOC), SMQ lack of efficacy/effect preferred terms, pregnancy, puerperium, and perinatal conditions (SOC) preferred terms, general disorders and administration site condition (SOC) preferred terms, and injury and poisoning and procedural complications (SOC).

These are in sync with the current categorization and classification of the World Health Organization: substandard, unregistered/unlicensed, and falsified. Substandard medical products—also called “out of specification” products—are authorized medical products that fail to meet necessary quality standards or specifications. Unregistered or unlicensed medical products that have not undergone evaluation or approval by the National or Regional Regulatory Authority (NRRA) for the market in which they are marketed or distributed are subject to permitted conditions under national or regional regulations and legislation. On the other hand, falsified medical products are the ones that deliberately or fraudulently misrepresent their identity and composition or source (World Health Organization, 2017; World Health Organization, 2022).

Nevertheless, the authors concentrated on the narrower and more definite falsified category and, therefore, selected the following reaction terms: counterfeit product administered, product counterfeit, product label counterfeit, product packaging counterfeit, suspected counterfeit product, adulterated product, product tampering, and suspected product tampering.

API or active pharmaceutical ingredient or active substance is defined according to the Directive 2001/83/EC of the European Parliament and of the Council as any substance or mixture of substances intended to be used in the manufacture of a medicinal product and that, when used in its production, becomes an active ingredient of that product intended to exert a pharmacological, immunological, or metabolic action with the view to restoring, correcting, or modifying physiological functions or to making a medical diagnosis (European Parliament and Council, 2016).

### Data analysis—deduplication and quality control

The EV data according to the latest guidance have a procedure for deduplication (Postigo et al., 2018; European Medicines Agency, 2020), and their data were used without searching for duplicates. In contrast, in the case of the FAERS, the data were cleaned to mitigate duplicate reporting. A total of 172 duplicates were removed from the data file. The basis for the identification of these duplicates was the product name, subject characteristics, such as sex, age, and weight, if the data was available, and the indication for the product or the potential ADR. Generally, if three parameters (age, product, and sex) were the same in two cases, we identified them as the same. The search, the extraction, and the cleaning of data were done separately by two researchers. For the analysis of the affected active pharmaceutical ingredients, when multiple products were listed in a case report, the first one was selected every time.

### Signal detection and statistical analysis

We used the publicly available OpenVigil 2 software to extract signals of drugs that were associated with ADRs indicative of counterfeit medicine in the FAERS database. In OpenVigil, data from 2004Q1 to 2022Q1 can be analyzed. We used the previously selected preferred terms. The program is presenting an ADR as putative whenever the followings are true: report count >3, PRR >2, and χ2 >4 (Böhm et al., 2021; Chiappini et al., 2022).

Unfortunately, access to other comprehensive electronic reaction monitoring reports such as EudraVigilance data analysis system (EVDAS) and the UMC VigiBase is only available for the marketing authorization holders (MAH) and regulatory agency members, therefore, could not be applied for EV data (Vogel et al., 2020).

For the EV dataset, we have calculated the input data from Supplementary File S1 (counterfeit related number of ADRs) and from the EV database (public search for all the ADRs by the defined API) and total ADRs until 2022Q2 (by 31 December 2021, the EudraVigilance database held a total of 12,530,776 individual cases). We have used the same threshold values as the OpenVigil (report count >3, PRR >2, and χ2 > 4) (European Medicines Agency, 2022b).

BM SPSS Statistics for Windows, version 28 (IBM Corp., Armonk, NY, United States) was applied for all descriptive analyses and to calculate pharmacovigilance signal measures in the EV dataset, including the reporting odds ratio (ROR), proportional reporting ratio (PRR), and relative reporting ratio (RRR) using Pearson’s chi-squared test. A *p*-value less than 0.05 was considered statistically significant.

## Results

**FIGURE 1:**
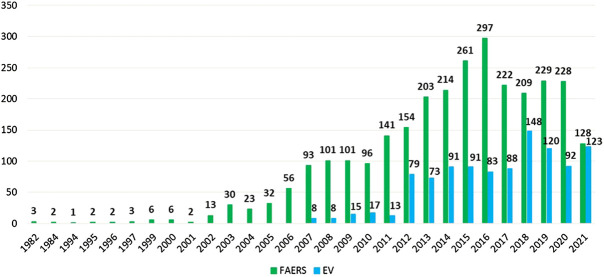
Number of cases identified in the FAERS and EudraVigilance databases per year.

A total of 5,253 cases in the FAERS (0.022% of 24,251,919) and 1,049 cases in the EV (0.023% of 4,512,023) were identified based on our search that can be related to the consumption of falsified medications. We have found cases from 1982 in the US database, while the first ADR potentially originated from a counterfeit product that was reported in 2007 to the EV database. As it can be seen in Figure 1, there has been a general increase in the reporting of ADRs in the last couple of years, slightly affected and declined by the COVID-19 pandemic.

**FIGURE 2:**
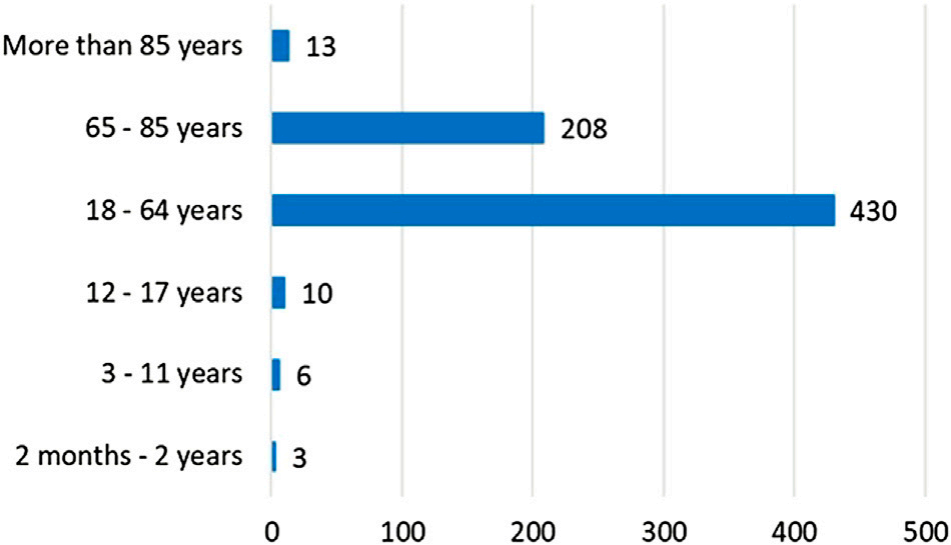
Age groups of cases identified in the EudraVigilance database (*n* = 670).

The two databases are collecting and presenting their data in a very similar way, although the exact age, the severity and geographical location, or even a literature reference cannot be extracted from the European one (see Table 1 and Figure 2). When we look at the main characteristics of patients affected with such ADRs, it can be seen that middle-aged men are more likely to be affected, as the mean age was 51.055 (SD: ±19.62) in the FAERS, and 64.18% of the cases were between 18 and 65 years in the EV, while 47.67% in the US database and 54.62% in the EV data were male consumers/patients. However, it must be noted that unfortunately, cases related to 1- and 2-month-old children and elderly (100 and 116 years old) were also present. In the FAERS dataset, more than 50% of the case listings (55.02%) did not specify the age. This result was 36.13% in the other one. Contrary, the sex is more specified in both reporting systems, as these data were only missing in 12.03% (FAERS) and in 8.96% (EV) of the cases. Statistical difference was found between the two databases related to the age groups (Pearson’s χ2, *p* < 0.001). For FAERS, the reports related to patients/consumers under 18 were 198, while for the EV, it was 19, and the difference was inverse for >65 years (FAERS: 79; EV: 221).

**TABLE 1:** Summary of the main characteristics of cases identified in the two pharmacovigilance databases.

	FAERS (1982–2021)	EV (2007–2021)
Total number of cases	5,253	1,049
Age range ratio		
Mean	51.055	–
Minimum	0 (1 month)	2 months—2 years
Maximum	116	More than 85 years
SD	19.262	–
Not specified	2,890 (55.02%)	379 (36.13%)
Gender ratio		
Female	2,117 (40.30%)	382 (36.42%)
Male	2,504 (47.67%)	573 (54.62%)
Not specified	632 (12.03%)	94 (8.96%)
Severity of outcome		
Non-serious	2,467 (46.96%)	142 (13.55%)
Serious	2,786 (53.04%)	906 (86.45%)
No. of death	183 (3.48%)	–
Congenital anomaly	3 (0.057%)	–
Disabled	114 (2.17%)	–
Hospitalized	458 (8.72%)	–
Life threatening	102 (1.94%)	–
Other outcomes	1,918 (36.51%)	–
Required intervention	8 (0.15%)	–
Geographical location		
Europe	500 (9.52%)	261 (24.88%)
United States	3,170 (60.35%)	–
Other	948 (18.05%)	788 (75.12%)
Not specified	635 (12.09%)	–
Reporter type		
Healthcare professional	1,222 (23.26%)	439 (41.85%)
Consumer	3,864 (73.56%)	610 (58.15%)
Not specified	167 (3.18%)	–
Literature references		
No events with references	223 (2.34%)	0 (0%)
No literature	44 (0.84%)	0 (0%)

When we look at the severity categories of the cases related to false medicinal products, there is a great contrast as in the FAERS. The ratio of serious and non-serious cases is almost 1 to 1 (46.96% and 53.04%), but in contrast, ADRs represented in the EV database are primarily (86.45%) severe. Also, 183 death cases were identified in the FAERS data.

After the analysis of the geographical origin of the reports, we have found that as FAERS is logically US specific, the counterfeit medicine ADR reports in the EV database were only sourced from the EU in 24.88%. Surprisingly, the consumer-related ADR reports in this field were much higher than the healthcare professional ones (73.56% and 58.5% compared to 23.26% and 41.85%, statistically significant, *p* < 0.001). In the FAERS listings of cases, we could analyze the country where the case occurred, and as can be seen in Table 2, the US represented almost 70% of the cases (68.62%), and only four countries had more than 100 cases in total, like China (396), Russia (166), the United Kingdom (115), and Brazil (113). With the help of the OpenVigil database, we calculated the number of ADRs with a strong relation to counterfeit medicines (report count >3, PRR >2, and χ^2^ > 4.) presenting another top list with United States, China, Germany, and the United Kingdom above 10 cases (in order 102, 37, 14, and 14). In the top 10, only one EU country was found (Germany). We could not extract similar data from the EV line listings.

**TABLE 2:** Top 10 countries with counterfeit-related ADRs in the FAERS database (1982–2021) and presumptive ADR in the OpenVigil database (2004Q1 to 2022Q1).

Country name	No. of events (N = 4,618)	%	No. of ADR^a^ (N = 192)
United States	3,169	68.62%	102
China	396	8.58%	32
Russia	166	3.59%	10
United Kingdom	115	2.49%	14
Brazil	113	2.45%	4
Mexico	89	1.93%	5
Germany	78	1.69%	14
Columbia	51	1.10%	4
Australia	51	1.10%	4
India	33	0.71%	3

a In OpenVigil, data from 2004Q1 to 2022Q1 can be analyzed. We used the previously selected preferred terms. The program is presenting an ADR as putative whenever the following are true: report count >3, PRR >2, and χ^2^ >4.

The detailed analysis of the cases gave us the opportunity to check the pharmaceutical companies behind the reports (see Table 3). In the US database, Pfizer was the first with 904 (19.02%) reports, while in the EV database, SANOFI-AVENTIS GROUPE was the leading company (160, 15.25%). In both databases, in the third place, the medicine agency report collections (FDA-CTU and EEA regulators) could be found.

**TABLE 3:** Top 10 pharmaceutical companies by the number of reports in the two pharmacovigilance databases.

Company name	Reported events in the FAERS (N = 4,752)	%	Company name	Reported events in the EV (N = 1,049)	%
Pfizer	904	19.02%	Sanofi-Aventis Groupe	160	15.25%
Eli Lilly and Co.	409	8.61%	Roche Products Limited	145	13.82%
FDA-CTU	263	5.53%	EEA Regulator	122	11.63%
Janssen	244	5.13%	Pfizer S.R.L.	89	8.48%
Johnson and Johnson	197	4.15%	Bristol-Myers Squibb Belgium	85	8.10%
Roche	196	4.12%	Eli Lilly and Company Limited	74	7.05%
Teva	138	2.90%	Bayer AG	73	6.96%
AbbVie	132	2.78%	Janssen-Cilag Limited	52	4.96%
Purdue	129	2.71%	Gilead Sciences International Limited	46	4.39%
Bristol-Myers Squibb	103	2.17%	Merck and Co., Inc.	42	4.00%

We also assessed the active pharmaceutical ingredients behind the ADR cases and compiled a top list. Two APIs were the same on each top 10 list: sildenafil and tadalafil. A strange new API in a counterfeit list is rivaroxaban, ranking fifth in the EV top 10 (see Tables 4, 5).

**TABLE 4:** Top 10 active pharmaceutical ingredients/products by the number of reports in the FAERS pharmacovigilance database.

Name of API/product	API	PRR (95%CI)	RRR (95%CI)	ROR (95%CI)	Number of cases for this drug
Sildenafil	Sildenafil	12.99 (11.57–14.58)	11.97 (10.67–13.44)	13.04 (11.61–14.64)	314
Tadalafil	Tadalafil	11.51 (9.98–3.27)	10.94 (9.49–12.62)	11.55 (10.02–13.33)	200
Oxycodone	Oxycodone	2.47 (2.14–2.86)	2.40 (2.07–2.77)	2.47 (2.14–2.86)	190
Alprazolam	Alprazolam	5.40 (4.61–6.31)	5.20 (4.45–6.09)	5.40 (4.62–6.32)	163
Fentanyl	Fentanyl	5.89 (5.03–6.89)	5.68 (4.85–6.65)	5.90 (5.04–6.91)	161
Viagra	Sildenafil	19.64 (16.39–23.54)	19.03 (15.88–22.80)	19.78 (16.50–23.70)	122
Dextroamphetamine	Dextroamphetamine	13.09 (10.73–15.96)	12.76 (10.47–15.55)	13.15 (10.78–16.03)	101
Amphetamine	Amphetamine	12.07 (9.84–14.81)	11.79 (9.61–14.46)	12.12 (9.88–14.87)	95
Orlistat	Orlistat	10,77 (8.69–13.34)	10.54 (8.51–13.06)	10.81 (8.73–13.39)	86
Emtricitabine	Emtricitabine	2.39 (1.81–3.16)	2.37 (1.80–3.14)	2.39 (1.81–3.16)	50

**TABLE 5:** Top 10 active pharmaceutical ingredients/products by the number of reports in the EV pharmacovigilance database.

Name of API/product	API	PRR (95%CI)	RRR (95%CI)	ROR (95%CI)	Number of cases for this drug
Clopidogrel	Clopidogrel	25.93 (21.19–31.73)	23.43 (19.17–28.65)	25.98 (21.23–31.79)	105
Bevacizumab	Bevacizumab	20.03 (15.77–25.46)	18.73 (14.77–23.82)	20.06 (15.79–25.49)	72
Insulin	Insulin	5.99 (4.65–7.74)	5.70 (4.42–7.35)	6.00 (4.65–7.74)	63
Sildenafil	Sildenafil	42.16 (32.47–54.73)	39.80 (30.67–51.65)	42.29 (32.58–54.90)	60
Rivaroxaban	Rivaroxaban	5.40 (4.12–7.09)	5.17 (3.95–6.79)	5.41 (4.12–7.09)	55
Entecavir	Entecavir	161.26 (119.79–217.10)	154.24 (114.59–207.58)	163.32 (121.31–219.87)	46
Pregabalin	Pregabalin	8.51 (6.21–11.68)	8.23 (5.99–11.28)	8.52 (6.21–11.69)	40
Tadalafil	Tadalafil	42.47 (30.83–58.51)	40.93 (29.71–56.37)	42.61 (30.93–58.69)	39
Abatacept	Abatacept	10.91 (7.01–17.00)	10.73 (6.89–16.70)	10.93 (7.02–17.01)	20
Tenofovir	Tenofovir	142.10 (91.06–221.75)	139.41 (89.35–217.53)	143.74 (92.11–224.30)	20

We used OpenVigil 2 to extract signals of drugs that were associated with ADRs indicative of counterfeit medicine in the FAERS database. This study detected 138 signals and several new APIs or products not represented in the literature. The identified top 10 based on the number of cases starts with sildenafil (*n* = 314; PRR, 12.99; ROR, 13.04; RRR, 11.97), tadalafil (*n* = 200; PRR, 11.51; ROR, 11.55; RRR, 10.94), and oxycodone (*n* = 190; PRR, 2.47; ROR, 2.14; RRR, 2.47). APIs and products such as alprazolam, fentanyl, Viagra, dextroamphetamine, amphetamine, orlistat, and emtricitabine were the followings. Compared to other APIs in the case of sildenafil counterfeit, ADR was reported more than 12 times as frequently [proportional reporting ratio (PRR) = 12.99]. The highest PRRs were identified for natural phospholipid product 1 (Survanta, *n* = 9; PRR, 196,33; 99.69–386.67; 95% CI), azithromycin (*n* = 4; PRR, 126,56; 46.39–345.26; 95% CI), natural phospholipid product 2 (Beractant, *n* = 10; PRR, 112,32; 59.60–211.65; 95% CI), and amfepramone (*n* = 3; PRR, 94,90; 29.98–300.33; 95% CI) an anti-obesity drug (now under investigation to withdraw from market in the EU, European Medicines Agency, 2022c), followed by minoxidil, benzoylecgonine, acetaminophen, levamisole, ketoconazole, and etizolam (the details are highlighted in the Supplementary Files S2,S3). 

With the EV data, 31 signals were identified by the disproportionality method. Based on the number of cases, clopidogrel (*n* = 105, PRR = 25.93, RRR = 23.43, ROR = 23.59, *p* < 0.001), bevacizumab (*n* = 72, PRR = 20.03, RRR = 18.73, ROR = 20.06, *p* < 0.001), and insulin (*n* = 63, PRR = 5.99, RRR = 5.70, ROR = 6.00, *p* < 0.001), while sildenafil in this database was fourth based on case numbers and sixth when checked by PRR (*n* = 60, PRR = 42.16, RRR = 39.80, ROR = 42.29, *p* < 0.001). The top 3 here are vardenafil (*n* = 16, PRR = 167.19; 101.71–274.84; 95% CI, RRR = 164.66; 100.17–270.66; 95% CI, ROR = 169.47; 103.09–278.60; 95% CI, *p* < 0.001), entecavir (*n* = 46, PRR = 161.26; 119.79–217.10; 95% CI, RRR = 154.24; 114.59–207.58; 95% CI, ROR = 163.32; 121.31–219.87; 95% CI, *p* < 0.001), and tenofovir (*n* = 20, PRR = 142.10; 91.06–221.75; 95% CI, RRR = 139.42; 89.35–217.53; 95% CI, ROR = 143.74; 92.11–224.30; 95% CI, *p* < 0.001) (the details are highlighted in Table 5).

## Discussion

The reporting of ADRs and ADRs identified in our study is quite similar in tendencies when we exclude the pandemic, and there are publications that have found no change in reporting (Dörks et al., 2021); however, there is a slight decrease in the number of falsified related cases, which can be derived from the fact that these cases are mainly hospital related and during the pandemic several institutions were reorganized to COVID-19 care and minimized other health care services (Moynihan et al., 2021).

Although the number of cases identified in the FAERS database is five times higher (5.01) than the cases reported in the EV, the ratio compared to all the reports in these databases is the same (0.022% and 0.023%). However, the difference between the two databases is presented in the number of identified signals as well (138 vs. 31). The reason behind the distortion represented in the geographical origin of cases reported to the EV can be linked to the phenomenon that falsified medicine case reporting is more common in non-EEA countries and that there is an obligation to report all serious ADR, while in EEA countries not just the reporting but the drug supply chain regulation is tightly controlled. Although we do not know the exact explanation, what can be seen is that in the case of other research studies not related to falsified medicine ADRs, we have also found the dominance of non-EEA reports (Ferreira et al., 2022; Rastogi et al., 2022).

It was a surprising finding that the majority of the reports originated from consumer reports, which contrasted with other ADR report analyses, such as in Germany, where 86.5% of ADR reports originated from healthcare professionals and 12.2% from non-healthcare professionals (Leitzen et al., 2021). In the case of the FAERS database, when Andreaggi et al. (2020) assessed the opioid-related cases, they found that it was more likely that an opioid-related ADR was reported by consumers compared to healthcare workers. The origin of the reports is similar to the review published by Rahman et al. (2018) as the United States, China, Russia, and the United Kingdom are represented in the incidents included in their work as well.

If we assess the pharmaceutical companies behind the reports, we have identified the same companies that can be found in the 2020 report from OECD/EUIPO. Pfizer, Roche, and Johnson and Johnson incorporated various anti-counterfeiting policies in their work (OECD/EUIPO, 2020).

Based on the review of 33 medicine quality safety studies by McManus D. and Naughton B.D. in 2020 (McManus and Naughton, 2020), the general and mixed prevalence of SF medicines for low-, middle-, and high-income countries are quite high (25%) and comparable with the result (28.5%) published in a 2013 review by Almuzaini et al. (2013). Another systematic review and meta-analysis focusing on low- and middle-income countries reported an overall prevalence of poor-quality medicines (studies that tested 50 samples or more) was 13.6% (Ozawa et al., 2018). According to the Pharmaceutical Security Institute (PSI), the global incidence of SF medicines has increased by almost 300 percent between 2011 and 2020, seeing the highest number in 2019 with 5,081 cases (in 2002, only 196 cases were in the database.) (Pharmaceutical Security Institute, 2022). According to our Pharmacovigilance data, we have identified more than 5,000 cases in almost 40 years in the FAERS and approximately 1,000 cases in a 14-year period. Although it is not comparable, as the cases presented in FAERS and EV are related to health damages requiring hospitalization. Also, the identification of these cases is not 100% and not efficient yet; furthermore, we did not include the falsified cases reported as product issues. That can be the reason for the discrepancies, and it is definite that multiple sources of data may have duplicates as well.

The SF medicine problem cannot be narrowed down just to sexual- and other performance-enhancing medications. Several other therapeutic categories and even life-saving medicines are affected (e.g., antibiotics and antimalarials, oncotherapeutics, biological drugs, and cardiovascular medicines) (Kelesidis and Falagas, 2015; Janvier et al., 2018; Ozawa et al., 2018; Venhuis et al., 2018; Eberle et al., 2020; Tesfaye et al., 2020; Świeczkowski et al., 2022). Therefore, the health effects or harms associated with SF medicines are also showing a wide range of possibilities, such as treatment failure, toxicity, and antimicrobial resistance, making it even harder to identify. Case studies published in the literature can help to understand the potential danger, such as bacterial infection and antimicrobial resistance (Chachere, 2005; Entezari et al., 2016) and wrong ingredients like in the case of sibutramine-containing products sold as orlistat products (US Food and Drug Administration, 2010). Tragically, everyone remembers the fake bevacizumab outbreak in the United States and in the EU, which led to tighter regulations like FMD and DSCSA (Kuehn, 2013; Blackstone et al., 2014; Taylor, 2019). What we can learn from the sampling studies is that tendencies and scenarios are more likely to happen. The two aforementioned reviews are quite helpful when we would like to assess the potential health consequences of SF medicines. These reviews show us the tendencies, such as the increasing quality studies performed in more and more middle- and high-income countries as well as the emerging anti-counterfeiting strategies implemented worldwide (e.g., unique identifiers and hubs for medicines and medical devices). The authors presented that quality assessment of these SF products showed that the most common issues were inadequate or excessive amounts of the active ingredient, no active ingredient, dissolution failure, wrong ingredient, and impurities. Although there are potential limitations to these findings, as these studies and their execution vary from country to country and are heavily affected by the opportunities, technologies available, and financial and human resource capacities at the time of the analysis (McManus and Naughton, 2020; Almuzaini et al., 2013; Hamilton et al., 2016; Tcheng et al., 2021; European Commission Delegated Regulation (EU) 2016/161, 2016). When we take a closer look at the adverse reactions or any other health damages presented in the literature, we are lacking information. A cornerstone publication is a review by Rahman M.S. et al. in 2018 (Rahman et al., 2018). The review found that based on the literature published, the actual prevalence of health damage related to SF medicines seems to show the minimal difference between developing and developed countries. It cannot be stated that the occurrence must be the same in every country, but it shows an increase in the awareness and identification of health problems with SF medicines in developed countries, such as the United States, Australia, the United Kingdom, etc. We are still far away from the realization of the total number of people affected by this global phenomenon. When we look at the therapeutic categories identified by Rahman et al. (2018), we can see the classic medications such as sedative-hypnotics, narcotics, and PDE-5 inhibitors, while in the case of developing countries, multiple cases of contaminated (e.g., diethylene glycol) analgesic and antipyretic medicines were reported. The quality product issues that have led to health damages were the following: no active ingredient, active ingredient in harmful amounts (e.g., glibenclamide), containing different active ingredients, or unacceptably high levels of contaminants (cyanide-laced or metal-laced products) (Rahman et al., 2018). It is an interesting trend that in the case of the falsification of biotechnological medicines, the products contain no active ingredient and are mainly inert substances like salt. It can mimic lyophilized peptide molecules (Janvier et al., 2018). Review by Rahman et al. (2018) found that therapeutic categories of falsified drugs that caused health damage and were published in the literature were mainly sedatives, hypnotics, narcotics, and drugs for sexual dysfunction in both developing and developed countries. In our study, in both databases, sildenafil and tadalafil were among the top 10 APIs, while alprazolam was just in the case of the FAERS top list. The unexpected API rivaroxaban in the EV top list represented in US and Mexican cases in 2021 and 2022 (White, 2022). The other APIs represented in our results such as anti-HIV, analgesic, insulin, and antiepileptic medicines are in line with previous publications (Rahman et al., 2018).

Another famous case was the contaminated heparin example in 2008 in the United States, resulting in 785 ADRs and 81 deaths (Blossom et al., 2008).

The general problem when we would like to assess the health impact of SF medicines is the fragmented and incomplete reporting in multiple systems. For example, infectology cases related to SF may be reported in national nosocomial surveillance systems, while ADRs are reported in pharmacovigilance systems and coded in the ICD system (Hohl et al., 2014; Li et al., 2017).

Also, the forensic and clinical toxicology reports can contain cases related to SF medicine consumption. New areas such as toxicovigilance and forensic pharmacovigilance can be valuable tools in order to identify and link these cases (Labadie, 2012; Bertrand et al., 2016; Fadlallah et al., 2016).

As we mentioned previously, the combination of several methods, the addition of systematic literature search, evidence-based medicine tools, and regionally collected ADR data, when assessing the potential health consequences of SF medicine use in real-time or retrospectively, can be the right direction in this research field (Chimirri et al., 2013).

The fragmentation is also present in the literature and research groups as there are limited academic cross-country collaborations as found by Sweileh et al. (2021).

In contrast, there are multiple collaborations at the international level like the World Intellectual Property Organization (WIPO), World Trade Organization (WTO), World Customs Organization (WCO), United Nations Office on Drugs and Crime (UNODC), and operations IMPACT and PANGEA, but their work is rarely published due to law enforcement and data privacy issues (Stukina et al., 2016). The tendencies that can be seen in pharmacovigilance, that single information sources are not enough, and multiple data sources are required for adequate ADR surveillance, can be applied to SF medicines as well (Hamilton et al., 2016). The potential application of other sources of medicine-related clinical data (like electronic medical record, EMR) combined with laboratory findings and financing codes (like ICD) is inhibited by the structure and accuracy of the data. To analyze ADRs from clinical narratives, further method developments, such as natural language processing (NLP) and other MI and Big Data approaches, are required. Also, the general financing pressure in the health care systems that reflects on health care documentation and coding makes it unreliable for evidence-based research. However, as it was highlighted in other research methods, the possible tendencies, with detailed information on everyday practice, can be seen in these results as well (Wang et al., 2018; Rey et al., 2022).

The authors think that the modified version of the tool to identify patients harmed by substandard and falsified medicines developed by Anđelković et al. (2017) can be a good choice for prospective real-world data collection. This method can be applied in an ambulatory care setting on clients with potential risk factors, as it contains five domains, such as health complaints; medical history; use of medicines; use of health care products; lifestyles relevant for exposure to SF medicines. The SF medicine watchlist was developed based on Medicrime drug seizures and national Official Medicines Control Laboratories (OMCL) working actively in the market surveillance programs. Medicines such as antibiotics, anti-obesity medicines, lifestyle products, anabolics, products against erectile dysfunction, and psychoanaleptics were included in the questionnaire (Anđelković et al., 2017; Venhuis et al., 2018).

The addition of updated product quality and SF medicine information available (e.g., from WHO Medical Product Alert) and ADR information related to SF medicines (e.g., from research studies like this) can help to find the population that should be screened with this easily applicable and validated tool. The causality and evidence of SF medicine consumption and the ADR require further method development with the combination of point-of-care testing of suspected laboratory parameters and indicator substance levels (e.g., benzodiazepines and sildenafil) with their metabolites from blood/urine samples, as well as the full analytical and microbiological analysis of the suspected product itself.

Although the pharmacovigilance databases are the cornerstone of post-marketing safety surveillance in the control of closed medicine supply chains, their operation is not without limitations. Generally, the under-reporting of suspected ADRs in spontaneous reporting systems is a major limitation, which is also true in the case of the counterfeit phenomenon. Not just the under-reporting, but the selective reporting can bias these data. The reasons for these were presented in detail in previous publications by Imman (1996) and Lopez-Gonzalez et al. (2009).

Biagi et al. (2013) described a method that can be implemented for enhancing ADR reporting, and it can be adapted to falsified and substandard medicine cases as well, such as the incorporation of regular training initiatives, especially in the hospital setting in order to raise the awareness of health care professionals (Ribeiro-Vaz et al., 2016).

Another quality control step can be the development of standardized data queries, similar to the Standardized MedDRA Queries (SMQs) that help to identify and retrieve potentially relevant individual case safety reports. The validation and reliability testing of our selected preferred terms (PTs) can be a good next step in this research field (MedDRA, SMQ Introductory Guide, 2016).

### Limitations

We acknowledge the limitations of our research. First and foremost, the data used have a general varying quality characteristic originating from the spontaneous reports (reporting bias). The causality of suspected ADRs and the flagged medication is not evaluated and can be related to other illnesses or concomitant medicine applications. Usually, the data on drug dose or the timing and duration of exposure relative to the event are not included in the reports. Moreover, the nature of spontaneous reporting as data fields are incorrectly completed, and multiple GDPR regulations affect what is available for analysis (e.g., age, previous medical conditions, country-specific information, and national authorization). Not just the details, but the entry of the MedDRA categories and classification can be incorrect as well. Another limitation is that the total number of ADRs in pharmacovigilance databases is affected by the authorization differences of medications in time and place. It is also known that there can be possible under-reporting (mild cases) and over-reporting (severe or “interesting” cases) in these databases. The current legislation of EV data collection has also affected our research, as the same ADR may be flagged by different healthcare professionals, including physicians, pharmacists, and nurses, or multiple ADRs may be presented for one individual, resulting in report duplications or triplications and making it almost impossible to eliminate this bias with manual analysis (Chiappini and Schifano, 2016; Hauben et al., 2017; Chiappini and Schifano, 2018). Further complications may arise when we would like to assess the total numbers, as cases can be presented in both of the databases, and the identification and elimination of duplicates between pharmacovigilance databases are almost impossible.

Although we have listed several limitations, this research is the first to present data related to definite counterfeit and falsified medicine-related ADR cases and to describe their characteristics and affected active pharmaceutical ingredients. With these results, we can add further data to support the identification of patient harm, such as the tool from Anđelković et al. (2017). Further potential usage of our initial research can be the basis of other retrospective and prospective data collection strategies for drug–drug interactions as well as to increase real-world evidence and knowledge.

## Conclusion

Pharmacovigilance datasets can be a great additional tool in the identification of real-world data related to substandard and falsified medicine cases. The potential risk groups of consumers and medications in the developed countries are also a valuable addition to the scientific literature and a helpful source of information for health care professionals. Further studies, standardization of reporting, and pharmacovigilance database analysis are recommended. The potential of data mining and various Big Data methods is inevitable in this field. These post-marketing data can be the cornerstone of a real-time, prospective monitoring of clinical cases and supply chain sampling studies as well.

## Data Availability

The original contributions presented in the study are included in the article/Supplementary Material; further inquiries can be directed to the corresponding author.
